# Production of CCL20 from lung cancer cells induces the cell migration and proliferation through PI3K pathway

**DOI:** 10.1111/jcmm.12781

**Published:** 2016-03-10

**Authors:** Beibei Wang, Lin Shi, Xiaoru Sun, Lingyan Wang, Xiangdong Wang, Chengshui Chen

**Affiliations:** ^1^ Department of Lung MedicineThe First Affiliated HospitalWenzhou Medical UniversityWenzhouChina; ^2^ Zhongshan Hospital Biomedical Research CenterShanghai Institute of Clinical BioinformaticsFudan University Center for Clinical BioinformaticsFudan University Shanghai Medical CollegeShanghaiChina

**Keywords:** lung, CCL20, cancer, PI3K, production, epithelium

## Abstract

Tumour inflammatory microenvironment is considered to play a role in the sensitivity of tumour cells to therapies and prognosis of patients with lung cancer. The expression of CCL20, one of the critical chemoattractants responsible for inflammation cells recruitment, has been shown overexpressed in variety of tumours. This study aimed at investigating potential mechanisms of CCL20 function and production in human non‐small cell lung cancer (NSCLC). Expression of CCL20 gene and protein in lung tissues of patients with NSCLC and NSCLC cells (A549) were determined. The interleukin (IL)‐1β‐induced signal pathways in A549 and the effect of CCL20‐induced A549 cell migration and proliferation were determined using migration assays and cell‐alive monitoring system. Mechanisms of signal pathways involved in the migration of CCL20 were also studied. We initially found that NSCLC tumour tissues markedly overexpressed CCL20 in comparison with normal lung samples. In addition, IL‐1β could directly promote CCL20 production in lung cancer cells, which was inhibited by extracellular signal‐regulated kinase (ERK)1/2 inhibitor, p38 mitogen‐activated protein kinase (p38 MARP) inhibitor or PI3K inhibitors. CCL20 promoted lung cancer cells migration and proliferation in an autocrine manner *via* activation of ERK1/2‐MAPK and PI3K pathways. Our data indicated that IL‐1β could stimulate CCL20 production from lung cancer cells through the activation of MAPKs and PI3K signal pathways, and the auto‐secretion of CCL20 could promote lung cancer cell migration and proliferation through the activation of ERK and PI3K signal pathways. Our results may provide a novel evidence that CCL20 could be a new therapeutic target for lung cancer.

## Background

The inflammation has been suggested to play a critical role in the development of cancer and contributes to most human cancers 1. Of them, lung cancer is the most commonly diagnosed malignancy and the leading cause of neoplasm death 2, associated with the location and severity of inflammation. Experimental evidence demonstrated that that the infection with non‐typeable Haemophilus influenza caused a chronic pulmonary disease‐like bronchial inflammation and accelerated the development of lung cancer 3. Clinical evidence indicated that the presence of chronic obstructive pulmonary disease could increase the risk of lung cancer up to 4.5‐fold 4.

Chemokine (C‐C motif) ligand 20 (CCL20), also known as casein kinase II beta subunit 4, macrophage‐inflammatory protein‐3a, liver and activation‐regulated chemokine, or exodus, was originally described responsible for the recruitment of dendritic cells 5. Chemokine (C‐C motif) receptor 6 (CCR6), a family of G protein‐linked 7‐transmembrane receptors, was identified as the highly specific receptor for CCL20. CCL20 was found to mediate the migration of inflammatory cells and epithelial cells, probably involved in the migration and metastasis of cancer cells in colorectal, prostate or pancreatic cancer 6, 7, 8. In functional assays, CCL20 stimulation of colorectal cancer cells led to phosphorylation of the p130cas, an adaptor/scaffolding protein associated with cytoskeletal and other focal adhesion proteins involved in adhesion and migration, as well as to increased proliferation and migration of the cancer cells *in vitro* 6, 9. Moreover, stimulation with CCL20 led to the activation of the ERK‐MAP kinase and Act pathways in colorectal cancer cells 6.

The selective function of CCL20, mediated by the specificity of ligand binding and the selective expression of CCR6 on specific immature dendritic cells, may play a critical role in dendritic cell‐associated airway in asthma 10. Th2 cytokines and ambient particulate matter stimulate the synthesis and release of CCL20 by airway epithelial cells 10.Recent studies demonstrated that CCL20 overexpressed in the lung tissue harvested from patients with non‐small cell lung cancer (NSCLC) 11. The increased production of CCL20 from adrenal glands might contribute to the selective recruitment of CCR6‐expressing cancer cells in lung cancer 12, although the mechanism by which CCL20 is involved in the formation of lung cancer remains unclear.

This study aimed to evaluate the hypothesis that lung cancer cells *per se* may secrete CCL20 to chemoattract the infiltration of inflammatory cells to the tumour tissue, responsible for the development of tumour inflammatory microenvironment. MAPK and phosphoinositide‐3‐kinas (PI3K) signalling pathways may be involved in CCL20 production in lung cancer. Interleukin (IL)‐1β was used in this study to stimulate CCL20 production from lung cancer cells and activate signalling pathways of MAPK and PI3K. Furthermore, chemoattractive roles of CCL20 in the tumour cell recruitment and proliferative roles of CCL20 in the tumour growth were investigated, and the involvement of ERK1/2‐MAPK and PI3K pathway in these processes was monitored.

## Materials and methods

### Reagents

Recombinant human IL‐1β and CCL20/MIP‐3α were purchased from PEPROTECH (Rocky Hill, CT, USA). PrimeScript RTreagent and SYBR Premix Ex Taq were purchased from TaKaRa Ltd. (Dalian, China). Human CCL20/MIP‐3 alpha quantikine ELISA kit was purchased from R&D (Shanghai, China). Anti‐CCL20/MIP‐3αantibody was purchased from Abcam (HK, China). Anti‐p44/42 MAPK(Erk1/2), anti‐phospho‐p44/42 MAPK(Erk1/2) (Thr202/Tyr204), anti‐p38 MAPK, anti‐phospho‐p38 MAPK (Thr180/Tyr182), anti‐SAPK/JNK, anti‐phospho‐SAPK/JNK (Thr183/Tyr185) and anti‐GAPDH were purchased from Cell Signaling Technology (Boston, MA, USA). ERK1/2 inhibitor PD98059 was purchased from Biovision Company (Mountain View, CA, USA). p38 MAPK inhibitor SB203580 was purchased from Selleckchem (Shanghai, China). NF‐kB inhibitor PDTC was purchased from Calbiochem (Darmstadt, Germany). SHBM1009 (a new PI3K/mammalian target of rapamycin inhibitor) was synthesized by Fudan University. Cell‐IQ live cell imaging platform was manufactured by Chipmantech (Tampere, Finland) and equipped in Zhongshan Hospital Biomedical Research Center, Fudan University, Shanghai, China.

### Cell culture

Human NSCLC cell line A549 cells were obtained from Center for Biomedical Research, Zhongshan Hospital. Cells were cultured in 12‐well plate with DMEM supplemented with 10% fetal bovine serum (FBS; Gibco, Thermo Fisher Scientific, Shanghai, China), 100 U/ml penicillin and 100 mg/ml streptomycin at 37°C in a 5% CO_2_, 95% air environment in humidified incubators.

### Lung tissue sampling and collecting

Fifteen patients with NSCLC underwent surgery at Zhongshan Hospital and were included in the present study after informed consent was obtained. Patients with preoperative radiation or/and chemotherapy were excluded. Under pathologist's supervision, 15 tumour samples and paired normal tumour‐adjacent samples were collected from surgically resected tissues. Time from clamping of NSCLC arterial supply to resection was controlled, and all samples were stored in liquid nitrogen until RNA and/or protein extraction.

### mRNA expression

Total RNA was obtained from tumour samples, paired normal tumour‐adjacent samples and A549 stimulated with the specified agents using TRIzol (Invitrogen, Carlsbad, CA, USA) according to the manufacturer's instructions. cDNA was synthesized with reverse transcriptase (TaKaRa) using the total RNA extracted. Real‐time PCR amplification was performed with SYBR Green (TaKaRa) on ABI 7000 PCR instrument (Eppendorf, Hamburg, Germany). The sequences of the forward and reverse PCR primers were 5′‐ggacctgacctgccgtctagaa‐3′ and 5′‐ggtgtcgctgttgaagtcagag‐3′ for GAPDH, 5′‐atttattgtgggcttcacacg‐3′ and 5′‐ccaagtctgttttggatttgc‐3′ for CCL20 respectively.

### Assays of CCL20 proteins

A549 cells were cultured in 12‐well plates for 24 hrs and then treated with vehicle or agents for additional 24 hrs. Supernatant levels of CCL20 were measured according to the manufacturer's instructions.

### Measurements of signal pathways

We investigated the signalling pathways responsible for the up‐regulation of CCL20 expression in A549 by IL‐1β. The phosphorylated and total amounts of ERK1/2, p38 MAPK, JNK and AKT were measured in A549 cells cultured without serum for 24 hrs and then treated with vehicle (controls) or IL‐1β at 1 ng/ml for 10, 20 or 30 min. Protein samples (50 μg) were mixed with one‐fourth volume of SDS sample buffer, boiled for 5 min. and then separated through 10% SDS‐PAGE gels. After electrophoresis, proteins were transferred to polyvinylidene fluoride (PVDF) membranes by electrophoretic transfer. Membranes were blocked in 5% bovine serum albumin for 2 hrs, rinsed and incubated with primary antibodies (diluted at 1:1000 or 1:2000) in TBS at 4°C overnight. Primary antibody was then removed by washing in TBS‐tween thrice, and labelled by incubating with 0.1 mg/ml peroxidase‐labelled secondary antibodies against rabbit for 2 hrs. Bands were visualized by electrochemiluminescence and exposed to X‐ray film following washing thrice in TBS‐tween. Band intensities were measured with Quantity One software (Bio‐Rad Laboratories, Hercules, CA, USA).

### Tumour cell migration assay

Cell migrations were assayed on A549 using the Transwell chamber inserts (8 μm pore size; Corning, NY, USA). A549 (10^4^) in 200 μl of serum‐free DMEM were incubated at the upper chamber. The Transwell inserts were placed in 24‐well plates containing 600 μl of DMEM containing 10% FBS with the specified agents. After incubation at 37°C in 5% CO_2_ for 12 hrs, cells migrated through the permeable membrane were fixed in paraformaldehyde, stained with Giemsa and counted under an upright light microscope at the origin magnification ×200. Each assay was done at least in triplicate.

### Real‐time measurements of cell wound‐healing and proliferation

The real‐time measurements of cell wound‐healing and proliferation were measured by the real‐time cell monitoring system, using a Cell‐IQ cell culturing platform (Chip‐Man Technologies, Tampere, Finland), equipped with a phase‐contrast microscope (Nikon CFI Achromat phase contrast objective with 10× magnification) and a camera (Nikon, Nikon Imaging (China) Sales Co., Ltd., Shanghai, China). The equipment was controlled by Imagen software (Chip‐Man Technologies). Images were captured at 5 min. intervals for 72 hrs. Analysis was carried out with a freely distributed Image software (Cell‐IQ Imagen v2.9.5c; McMaster Biophotonics Facility, Hamilton, ON, Canada), using the Manual Tracking plug‐in created by Fabrice Cordelieres (Institut Curie, Orsay, France). Cell‐IQ system uses machine vision technology to monitor and record time‐lapse data, and it can also analyse and quantify cell functions and morphological parameters. The movement of each individual cell was measured in the image field by metering the distance of cell movement.

### Statistical analysis

Data were expressed as mean ± S.E., and were evaluated using anova with LSD test for multiple comparisons and Student's *t*‐tests for two groups. Increased rates of total cell number and differentiation were calculated as the following: Rate (%) = (value at each time‐point − value of primary seeding cells)/value of primary seeding cells × 100. Cell movement was calculated as the mean of the distance of every cell moving between two images (5 min. interval). *P* < 0.05 was considered as statistically significant.

## Results

**Figure 1:**
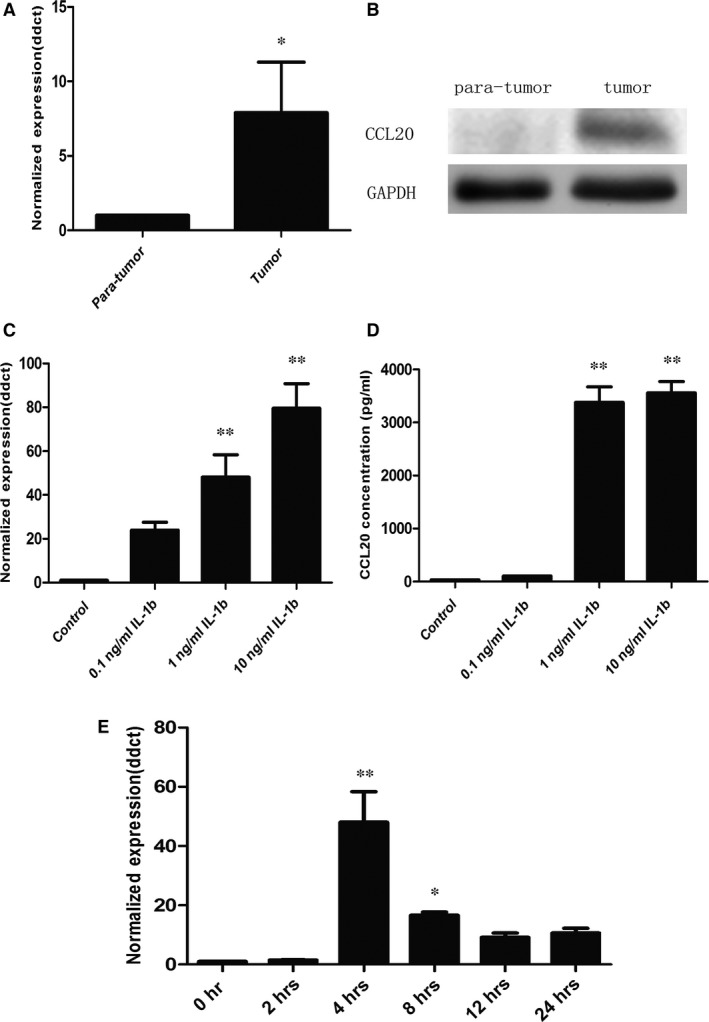
Expression of CCL20 in NSCLC. (**A**)RT‐PCR analysis of CCL20 mRNA expression in 10 freshly collected NSCLC tissues; (**B**) Western blotting analysis of CCL20 protein expression in 10 freshly collected NSCLC tissues; (**C**) RT‐PCR analysis of CCL20 expression in total mRNA harvested from A549 cells challenged with PBS (controls) or IL‐1β at 0.1, 1 or 10 ng/ml for 4 hrs; (**D**) ELISA assay of CCL20 expression in the supernatant harvested from A549 cells challenged with PBS (controls) or IL‐1β at 0.1, 1 or 10 ng/ml for 24 hrs; (**E**) RT‐PCR analysis of CCL20 expression in total mRNA harvested from A549 cells challenged with IL‐1β at 1 ng/ml from 0 to 24 hrs. Data were presented as mean ± S.E. and each group has at least six measurements (*P < 0.05, **P < 0.01).

**Figure 2:**
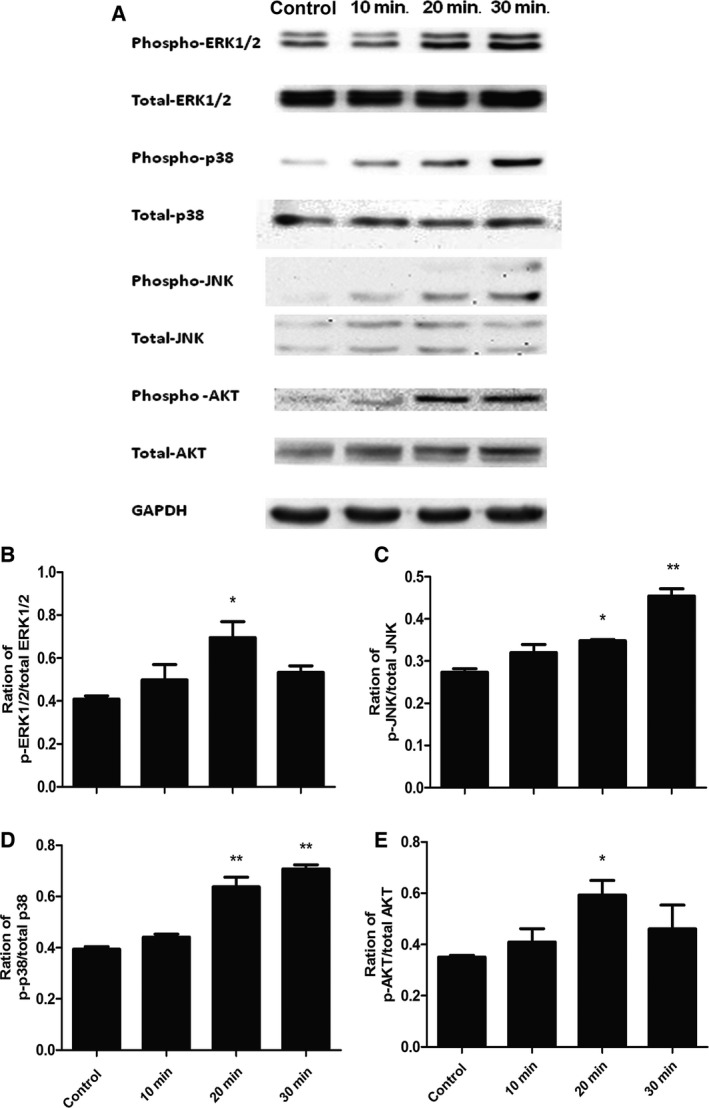
Activation of ERK1/2, p38, JNK and AKT induced by IL‐1β in lung cancer cells (A549 cells). (**A**) Phosphorylated and total amounts of ERK1/2, p38 MAPK, JNK and AKT measured by Western blot in A549 cells; Ratio of p‐ERK1/2/total ERK1/2 (**B**), p‐p38/total p38 (**C**), p‐JNK/total JNK (**D**) and p‐AKT/total AKT (E) in A549 cells. Starved cells were challenged without (control) or with IL‐1β at 1 ng/ml for 10, 20, 30 min. Data were presented as mean ± S.E. and each group has at least six measurements (*P < 0.05, **P < 0.01).

The expression of CCL20 mRNA and protein isolated from the lung cancer tissue was significantly higher than that from the normal lung tissue (Fig. 1A and B, *P* < 0.05), respectively. About 80% of patients with NSCLC had the overexpression of CCL20 proteins in the lung cancer tissue. We found that IL‐1β cold stimulate the significant overexpression and production of CCL20 mRNA in A549 cells (Fig. 1C) and proteins (Fig. 1D) in the supernatant from the dose of 1.0 ng/ml at 4 and 24 hrs, respectively, as compared with those stimulated with vehicle (*P* < 0.01). Interleukin‐1β‐stimulated expression of CCL20 mRNA significantly increased at both 4 and 8 hrs (*P* < 0.01 and 0.05, respectively, Fig. 1E). Figure 2A demonstrated the changes of total and phosphorylated ERK1/2, AKT, p38MARK and JNK of A549 cells stimulated with vehicle or IL‐1β. The amount of phosphorylated ERK1/2 (Fig. 2B) and AKT (Fig. 2E) increased significantly at 20 min. after the treatment with IL‐1β, while the amount of phosphorylated JNK (Fig. 2C) and p38MAPK (Fig. 2D) increased at 20 and 30 min.

**Figure 3:**
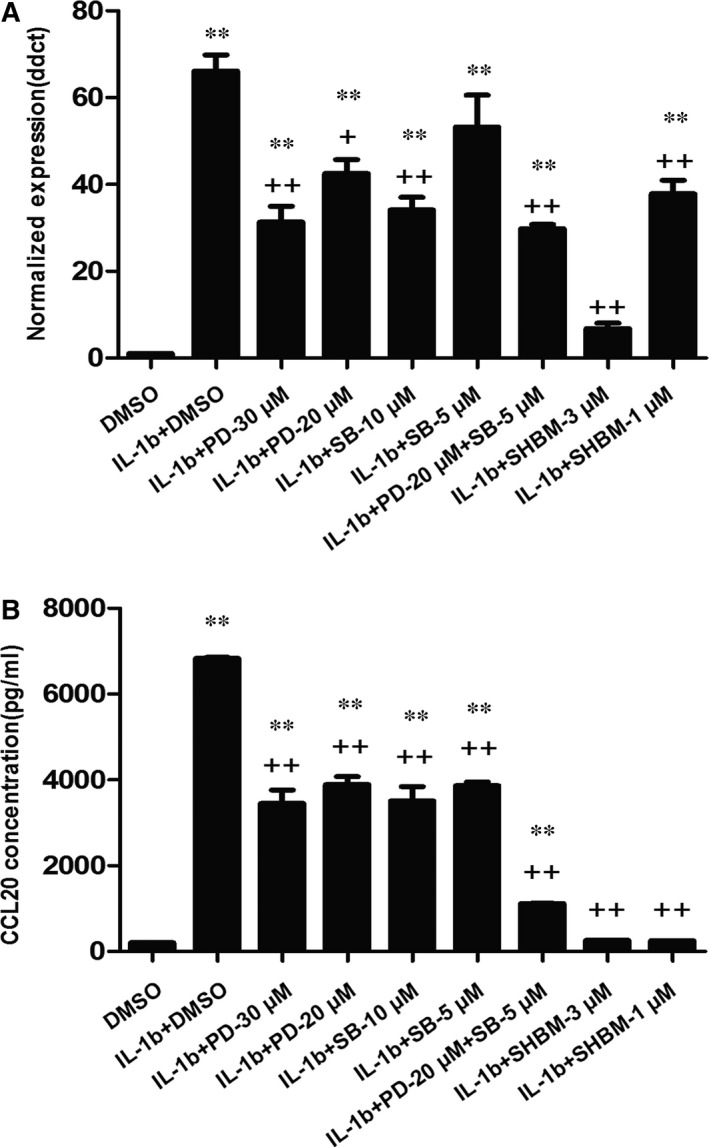
The effect of ERK1/2, p38 MAPK and AKT inhibitors on IL‐1β‐induced CCL20 production in A549 cells. Cells were pre‐treated with or without inhibitors of ERK1/2 (PD98059), p38‐MAPK (SB202190) and AKT (SHBM1009) for 1 hr and stimulated with IL‐1β (1 ng/ml), and the total mRNA was harvested in 4 hrs for RT‐PCR analysis (**A**) or the conditioned medium was collected in 24 hrs for ELISA assay (**B**). ** stands for *P*‐values less than 0.05 and 0.01, as compared with cells only with DMSO, and ++ stands for *P*‐value less than 0.05 and 0.01, as compared with IL‐1β and DMSO respectively. Data were presented as mean ± S.E. and each group has at least six measurements.

The expression of CCL20 mRNA in cells treated with PD98059 at 20 and 30 μM, SB203580 at 10 μM, the combination of PD98058 at 20 μM and SB203580 at 5 μM, or SHBM1009 at 1 μM was significantly lower than those treated with vehicle 4 hrs after IL‐1β stimulation, respectively; while still significantly higher than those without IL‐1β (*P* < 0.05 or less, respectively, Fig. 3A). The expression of CCL20 mRNA in cells treated with SHBM1009 at 3 μM was significantly lower than those treated with vehicle or other inhibitors 4 hrs after IL‐1β stimulation (*P* < 0.01 respectively). Supernatant levels of CCL20 proteins in cells treated with PD98058, SB203580 or both, were significantly lower than those with vehicle 24 hrs after IL‐1β stimulation, and higher than those without IL‐1β (*P* < 0.01, respectively, Fig. 3B). Levels of CCL20 proteins in cells treated with the combination of PD98058 and SB203580 at the low dose were significantly lower than those treated with either of them alone at the low and high does (*P* < 0.01). Levels of CCL20 proteins in cells treated with SHBM1009 at 1 and 3 μM were significantly lower than those treated with vehicle or other inhibitors, while not significantly different from cells without IL‐1β stimulation.

**Figure 4:**
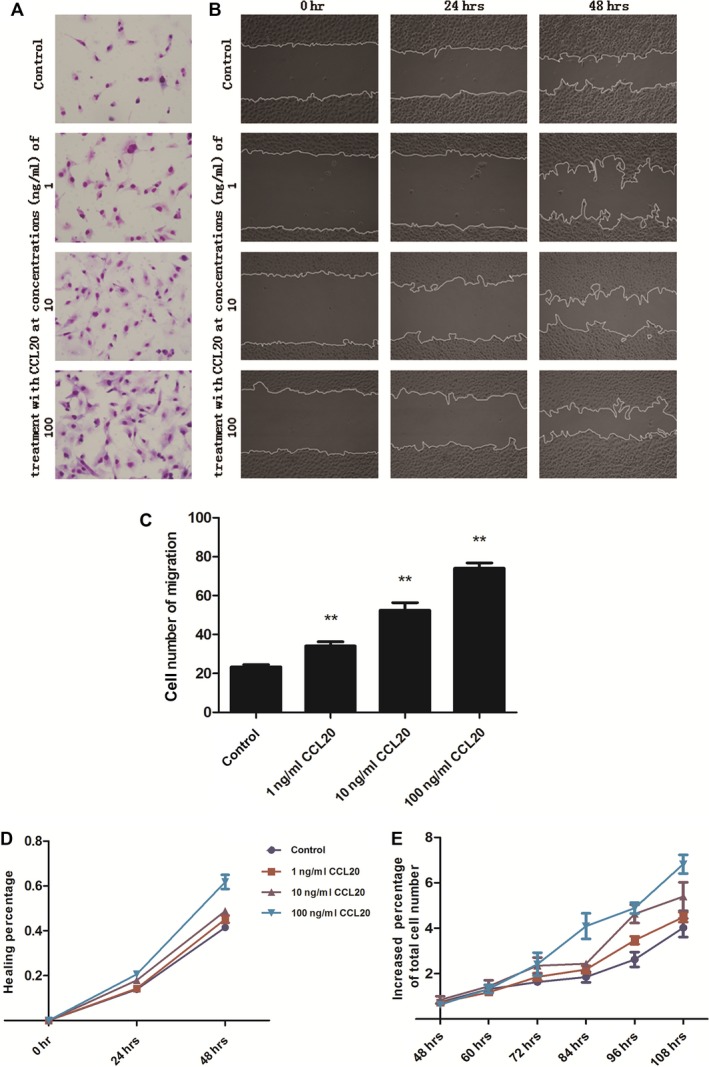
CCL20 induces A549 chemotaxis and proliferation. (**A** and **C**) Effect of extraneous recombinant CCL20 on the chemotaxis of A549 measured by transwell assays, A549 cells were incubated at the upper chamber with FBS free DMEM and extraneous recombinant CCL20 (1, 10, or 100 ng/ml) was added into the lower compartment with complete DMEM; (**B** and **D**) effect of extraneous recombinant CCL20 on the chemotaxis of A549 measured by wound‐healing assays in Cell‐IQ Alive Image Monitoring System, A549 cells were challenged with extraneous recombinant CCL20 (1, 10 or 100 ng/ml) in FBS free DMEM; (**E**) effect of extraneous recombinant CCL20 (1, 10 or 100 ng/ml) on the proliferation of A549 measured by Cell‐IQ Alive Image Monitoring System. ** stands for *P*‐values less than 0.05 and 0.01, as compared with control, data were presented as mean ± S.E. and each group has at least three measurements.

The chemoattractive function of CCL20 on NSCLC cell line A549 was measured by the cell migration assays in the transwell system and cell wound‐healing assays in the real‐time cell‐monitoring system. The number of migrated cells significantly increased 24 hrs after the stimulation with extraneous recombinant CCL20 at doses of 1, 10 or 100 ng/ml, which was confirmed by cell staining (Fig. 4A and C). This was also confirmed by the measurement of healing percentage in the wound‐healing assays. CCL20 at 10 and 100 ng/ml significantly increased the healing percentage of A549 cells at both 24 and 48 hrs (*P* < 0.05 or less, respectively, Fig. 4B and D). Figure 4E demonstrated the effect of CCL20 stimulation on A549 cells proliferation. The increased percentage of total cell number significantly increased after IL‐1β stimulation at different doses, as compared with those stimulated with vehicle (*P* < 0.05 or less).

**Figure 5:**
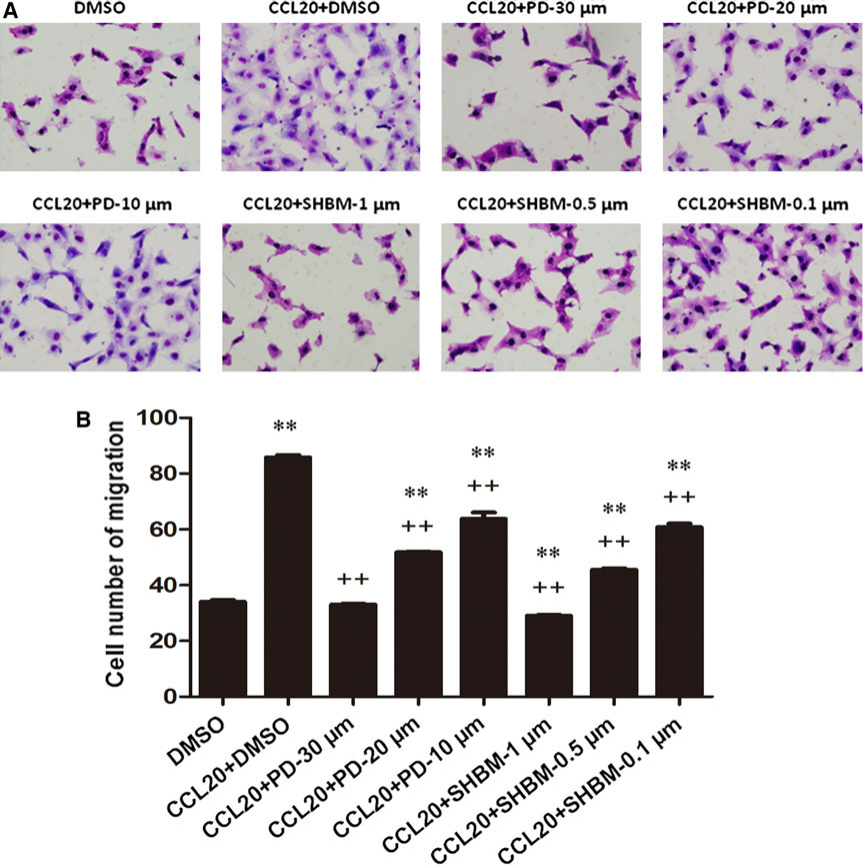
Roles of ERK1/2 and AKT signal pathways in the chemotaxis of CCL20 on A549. Effects of inhibitors of ERK1/2(PD98059) and AKT(SHBM1009) on CCL20‐induced migration of A549 measured by transwell assays. A549 cells challenged with DMSO or inhibitors were incubated at the upper chamber; extraneous recombinant CCL20 at 100 ng/ml was added into the lower compartment. ** stands for *P*‐values less than 0.05 and 0.01, as compared with cells only with DMSO, and ++ stands for *P*‐value less than 0.05 and 0.01, as compared with CCL20 and DMSO respectively. Data were presented as mean ± S.E. and each group has at least three measurements.

**Figure 6:**
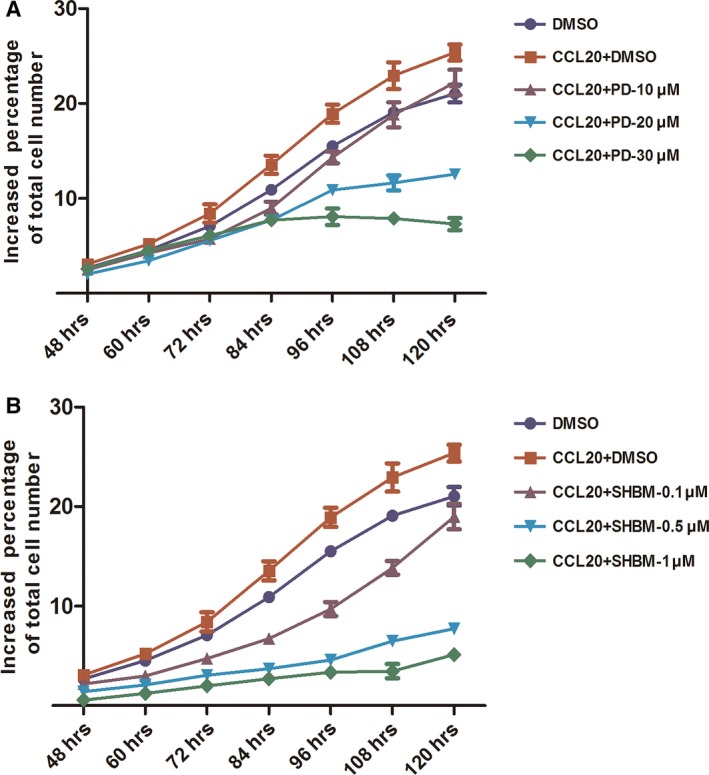
Roles of ERK1/2 and AKT signal pathways in the proliferation of CCL20 on A549. Effects of inhibitors of ERK1/2(PD98059) (**A**) and AKT(SHBM1009) (**B**) on CCL20‐induced proliferation of A549 cells measured by Cell‐IQ Alive Image Monitoring System. Data were presented as mean ± S.E. and each group has at least three measurements.

Figure 5 demonstrated that inhibitory roles of ERK1/2 and PI3K signal pathway inhibitors in CCL20‐induced A549 cells chemotaxis. The ERK1/2 inhibitor PD98059 and PI3K inhibitor SHBM1009 were pre‐incubated with cells in transwell system, followed by the stimulation of CCL20 at 100 ng/ml. The treatment with PD98059 at 10, 20 or 30 μM and SHBM1009 at 0.1, 0.5 or 1 μM significantly prevented CCL20‐induced cell migration in a dose‐dependent pattern (Fig. 5). CCL20‐increased A549 proliferation was significantly inhibited by both PD98059 and SHBM1009 at all concentrations in a dose‐dependent pattern, as shown in Figure 6A and B.

## Discussion

Chemokines have been suggested to play an important role in the formation of cancer microenvironment and be responsible for the migration and infiltration of both inflammatory cells and cancer cells 13. The expression of CCL20 is normally low on keratinocytes, pulmonary or intestinal epithelial cells, while could increase obviously after the activation of pro‐inflammatory signals by primary cytokines (*e.g*. TNF‐α) or toll‐like receptor agonists originating from microbes 5. CCL20 is the unique chemokine ligand for the CC‐chemokine receptor CCR6, a receptor which is mainly expressed on the certain staged cells, for example,. on the part of immature dendritic cells rather than CD14‐positive dendritic cell precursors or mature dendritic cells 14, different from the promiscuity of most chemokines with their receptors. Dendritic cells were attracted to the established tumours in the artificial accumulation of CCL20 and could suppress tumour growth 15, while tumour cells transfected with CCL20 could increase the intratumoural infiltration of immature dendritic cell and the tumour growth, and decrease immunogenicity 16. It was found that human breast carcinomas and NSCLC had high levels of CCL20 expression 17, 18.

This study provided solid evidence that CCL20 overexpressed in human lung cancer tissues and cells, which may act as the initiator for the secondary inflammatory responses in the development of the cancer microenvironment. The findings that CCL20 accelerated the migration and growth of cancer cells indicate that CCL20 may play the role in the maintenance of tumour growth and reconstruction of tumour tissues. It is also possible that inflammatory mediators‐induced overproduction of CCL20 from NSCLC cells and other cells may increase the resistance of cancer cells to therapies, the metathesis of cancer cells or the cell capability of residing or survival even in the new location.

It was found that the expression and production of IL‐1, especially secreted IL‐1β, increased during the tumour progression and were directly correlated with poor prognoses 19, 20. Previous findings demonstrated that CCL20 could be overexpressed and overproduced by lung cancer cells 8. Our results indicate that lung cancer cells may produce CCL20 in autocrine and paracrine manners to attract tumour‐associated macrophages and tumour cells. Studies also have showed that cytokines (*e.g*. TNF‐α) could stimulate the production of CCL20 5, and we have validated the stimulation effect of TNF‐α on A549, which was much slighter than IL‐1β.

The production of CCL20 regulated by inflammatory cytokines was suggested to be regulated by the activation of ERK1/2 and/or p38 MAPK pathways in human bronchial epithelial cells 10. Phosphoinositide‐3‐kinase pathway is widely distributed and closely related to cytokine production and cancer development 21, although the involvement of the PI3K pathway on CCL20 production remains unclear. The data from the present study demonstrated that multiple signal pathways might be involved in the regulatory mechanism of IL‐1β‐induced CCL20 overexpression and production. Of those intracellular signal pathways, the MAPK‐PI3K pathway in lung cancer cells might play the independent and necessary role in the regulation of CCL20 production during the formation of inflammatory microenvironment. It was suggested that pro‐inflammatory cytokines like IL‐1β induce the production of CCL20 through the NF‐kB regulation, since the production of CCL20 was suppressed in the presence of the mutant IkB protein 22.

However, our results showed that NF‐ kB inhibitor PDTC did not affect IL‐1β‐induced CCL20 production in A549 cells (Fig. S1), which may be because of the variation in cell types and inhibition approaches. Further studies are needed to investigate the roles of those pathways in the development and persistence of tumour‐associated inflammatory environment. We suppose that CCL20 may promote cancer progression by a direct action on cancer cells or establishing a microenvironment that suppresses specific anti‐tumour response. The fact that CCL20 could directly enhance the migration of A549 cells indicates that CCL20 could contribute to the metastasis of NSCLC, re‐orientate the direction of cell movements or the formation of local inflammatory microenvironment. It was found that patients developed the adrenal metastasis after resections of primary NSCLC and had high levels of CCL20 in adrenal glands responsible for the selective recruitment of CCR6‐expressing cancer cells from the lung cancer 12.

The activation of ERK–MAP kinases and Akt has been suggested to play an important role in cell migration and proliferation 21. CCL20 could stimulate the phosphorylation of ERK1/2, AKT or p130 cas, an adaptor/scaffolding protein, associated with cytoskeletal and other focal adhesion proteins involved in adhesion and migration in colorectal cancer cells 6, 9. Our data demonstrated that intracellular ERK1/2 or PI3K might be involved in the regulation of CCL20‐induced A549 cells migration and proliferation. It was reported that CCL20‐induced breast epithelial cells migration and growth were mediated by matrix metalloproteinases‐9 (MMP‐9) 23, while our data showed that CCL20 stimulated slight production of MMP‐9 in A549 cells (as shown in Fig. S1). The CCL20–CCR6 axis was found to mediate the migration of circulating T‐regulatory cells into the tumour microenvironment, leading to the tumour progression and poor prognosis in patients with hepatocellular carcinoma 24. FOXP3^+^Tregs appear normally in the bronchial epithelium but richly gathered in NSCLC cells and tumour‐infiltrating lymphocytes. It is suspicious that high concentrations of CCL20 in the lung tumour environment increase tumour progression *via* attracting those T cells, functioning as an immune escape mechanism 25.

## Conclusions

**Figure 7:**
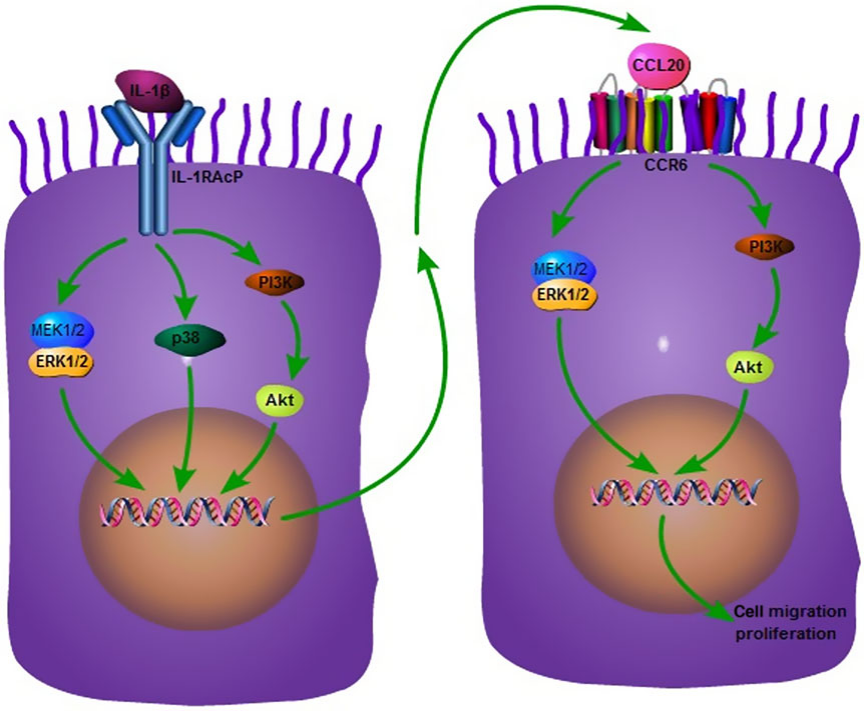
Proposed mechanism of CCL20 production and function in human non‐small cell lung cancer. Tumour cells *per se* act as the initiator, receptor or promoter of CCL20 responsible for the development and formation of inflammatory microenvironment in lung cancer as well as the transformation and growth of malignancy.

Interleukin‐1β could directly stimulate CCL20 production of lung cancer cells through the activation of MAPKs and PI3K signal pathways, and the auto‐secretion of CCL20 could promote lung cancer cells migration and proliferation through the activation of ERK and PI3K signal pathways (Fig. 7). Therefore, CCL20, which is up‐regulated in response to inflammatory stimuli, may have an important role in the development and progression of inflammatory microenvironment in lung cancer.

## Supporting information


**Figure S1** The effect of NF‐kB inhibitor on IL‐1β‐induced CCL20 secretion in A549 cells. Cells were pre‐treated with or without NF‐kB inhibitor PDTC for 1 hr and stimulated with IL‐1β (1 ng/ml), and the total mRNA was harvested in 4 hrs for RT‐PCR analysis (**A**) or the conditioned medium was collected in 24 hrs for ELISA assay (**B**). Data were presented as mean ± S.E. and each group has at least six measurements.

**Figure S2** The effect of CCL20 on MMP‐9 production in A549 cells. RT‐PCR analysis of MMP‐9 expression in total mRNA harvested from A549 cells challenged with PBS (controls) or CCL20 at 1, 10 or 100 ng/ml for 4 hrs. Data were presented as mean ± S.E. and each group has at least six measurements (*P < 0.05).
