# Supplementary figures and images for: Production of CCL20 from lung cancer cells induces the cell migration and proliferation through PI3K pathway
Source: J Cell Mol Med. 2016 Mar 10;20(5):920–9. doi: 10.1111/jcmm.12781 (PMC4831357; doi:10.1111/jcmm.12781)

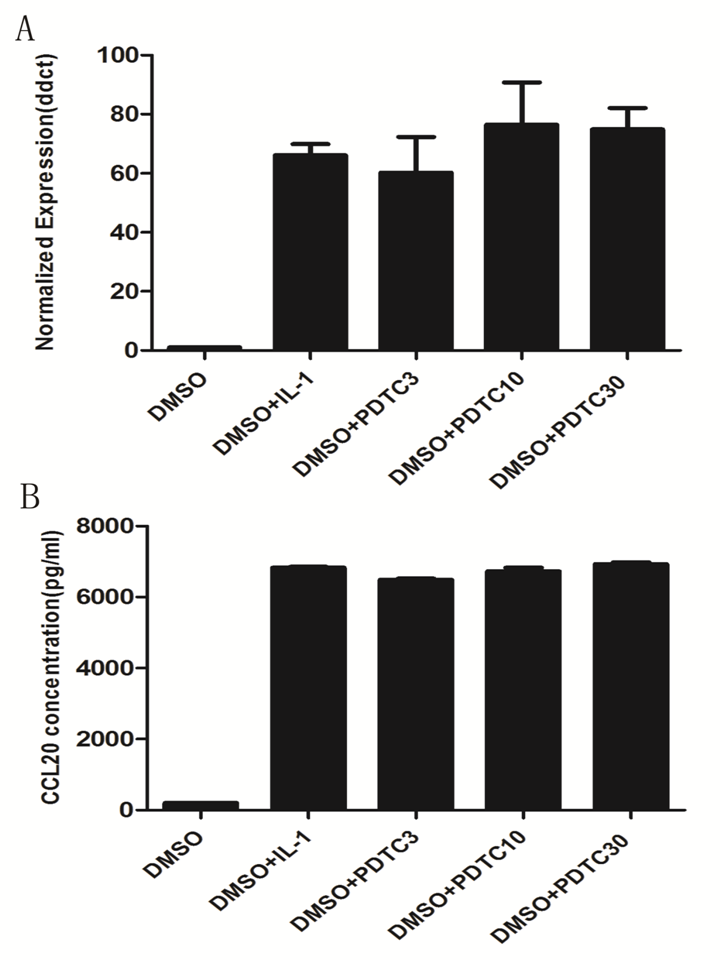

Supplement: Supplementary file 1 — Figure S1 The effect of NF‐kB inhibitor on IL‐1β‐induced CCL20 secretion in A549 cells. Cells were pre‐treated with or without NF‐kB inhibitor PDTC for 1 hr and stimulated with IL‐1β (1 ng/ml), and the total mRNA was harvested in 4 hrs for RT‐PCR analysis (A) or the conditioned medium was collected in 24 hrs for ELISA assay (B). Data were presented as mean ± S.E. and each group has at least six measurements. [file JCMM-20-920-s001.TIF]

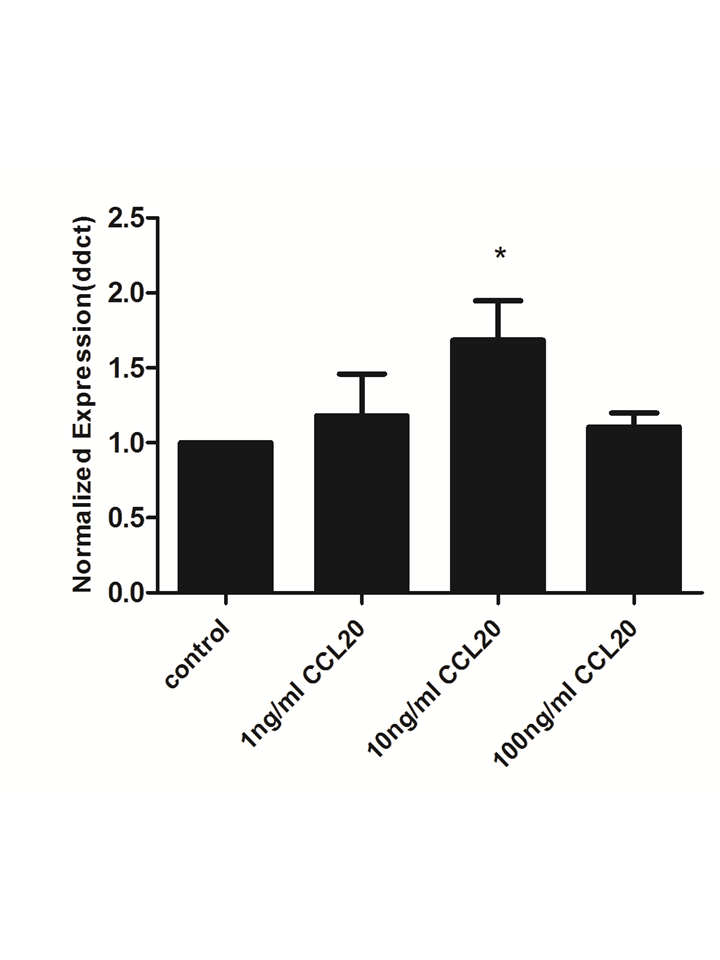

Supplement: Supplementary file 2 — Figure S2 The effect of CCL20 on MMP‐9 production in A549 cells. RT‐PCR analysis of MMP‐9 expression in total mRNA harvested from A549 cells challenged with PBS (controls) or CCL20 at 1, 10 or 100 ng/ml for 4 hrs. Data were presented as mean ± S.E. and each group has at least six measurements (*P < 0.05). [file JCMM-20-920-s002.TIF]
